# Rapid divergence of the male reproductive proteins in the *Drosophila dunni* group and implications for postmating incompatibilities between species

**DOI:** 10.1093/g3journal/jkab050

**Published:** 2021-02-18

**Authors:** Tom Hill, Hazel-Lynn Rosales-Stephens, Robert L Unckless

**Affiliations:** The Department of Molecular Biosciences, University of Kansas, Lawrence, KS 66045, USA

**Keywords:** *Drosophila*, hybrid incompatibilities, genome evolution, seminal fluid proteins

## Abstract

Proteins involved in post-copulatory interactions between males and females are among the fastest evolving genes in many species, usually attributed to their involvement in reproductive conflict. As a result, these proteins are thought to often be involved in the formation of postmating-prezygotic incompatibilities between species. The *Drosophila dunni* subgroup consists of a dozen recently diverged species found across the Caribbean islands with varying levels of hybrid incompatibility. We performed experimental crosses between species in the *dunni* group and see some evidence of hybrid incompatibilities. We also find evidence of reduced survival following hybrid mating, likely due to postmating-prezygotic incompatibilities. We assessed rates of evolution between these species genomes and find evidence of rapid evolution and divergence of some reproductive proteins, specifically the seminal fluid proteins. This work suggests the rapid evolution of seminal fluid proteins may be associated with postmating-prezygotic isolation, which acts as a barrier for gene flow between even the most closely related species.

## Introduction

Numerous groups of recently diverged species have been used to study speciation across multicellular taxa (Coyne and Orr 1989; McKinnon and Rundle 2002; Glor *et al.* 2005; Kitano *et al.* 2009; Brekke and Good 2014). These studies find an array of complex relationships between species caused by varying levels of divergence across their genomes, including premating isolation and reinforcement, postmating-prezygotic isolation and postzygotic isolation (Coyne and Orr 2004; Orr 2004; Presgraves 2007; Matute *et al.* 2010; Moyle and Nakazato 2010; Payseur and Rieseberg 2016). Recently diverged species with incomplete reproductive barriers prove to be more useful for understanding how new species evolve (Coyne and Orr 1989; Butlin *et al.* 2012). These species groups can be used in QTL studies to identify loci that contribute to the reduced fitness of hybrids (Howard *et al.* 2002; Noor *et al.* 2007; Kitano *et al.* 2009), or to identify genes that may be involved in the early stages of reproductive isolation, such as those causing incompatibilities in the heterogametic sex (known as Haldane’s Rule when incompatibilities in the heterogametic sex are in excess) (Haldane 1922; Coyne and Orr 1989; Orr 1995; Gavrilets and Waxman 2002; Coyne and Orr 2004; Phadnis and Orr 2009; Orr 1997).

Several studies have also highlighted that proteins transmitted in the seminal fluid to the female reproductive tract may be a major cause of postmating-prezygotic isolation (Bono *et al.* 2015; Ahmed-Braimah *et al.* 2017; 2020; Rowe *et al.* 2020). Further, previous work has highlighted that post-mating-prezygotic barriers are a major cause of incompatibilities in incompletely reproductively isolated species (Gavrilets and Waxman 2002; Coyne and Orr 2004; Andres *et al.* 2008; Gourbière and Mallet 2010; Larson *et al.* 2012; 2013; Ahmed-Braimah 2016; Turissini *et al.* 2018; Castillo and Moyle 2019; Matute *et al.* 2020) and form more rapidly than other types of incompatibilities (Turissini *et al.* 2018). 

Barriers to hybridization have also been examined in different *Drosophila* species groups, finding varying levels of divergence, and in some cases the mechanisms for isolation between species (Patterson 1947; Grant 1983; Coyne and Orr 1989; Presgraves 2007; Miller *et al.* 2010; Matute and Ayroles 2014; Ahmed-Braimah 2016; Turissini *et al.* 2018; Matute *et al.* 2020). Some studies, focusing on the effects of heterospecific matings on females, have found drastic changes in the females, including the swelling of the reproductive tract (Patterson 1947) and the activation of stress response pathways (Bono *et al.* 2011; Ahmed-Braimah *et al.* 2020). This is likely due to antagonistic interactions between male seminal fluid proteins and the heterospecific female tract (Knowles and Markow 2001; Bono *et al.* 2011; Ahmed-Braimah *et al.* 2017), and such pressure may drive the evolution of reinforcement (Coyne and Orr 2004; Turissini *et al.* 2018).

The *Drosophila dunni* subgroup is found within the *cardini* group in the *Drosophila* subgenus (Supplementary Figure 1) (Heed 1962). This species group diverged across the Caribbean islands, creating endemic populations each on a different island or set of islands (Heed 1962; Hollocher *et al.* 2000; Wilder and Hollocher 2003). Despite their geographic isolation from each other, species are still able to hybridize (to varying levels of success) and are a useful species group for understanding several traits, such as the evolution of pigmentation or reproductive isolation (Stalker and Streisinger 1953; Hollocher *et al.* 2000; Wilder and Hollocher 2003). In some crosses, these hybrid offspring show evidence of Haldane’s rule (Haldane 1922; Orr 1997), with crosses producing only female offspring, or sterile male offspring (Heed 1962).

Here we performed experimental crosses in the *dunni* group and found that in some crosses, heterospecific matings reduce female survival compared to conspecific matings, potentially caused by an insemination reaction-like effect (Patterson 1947). Using a combination of long-read and short-read sequencing, we assembled the genomes of four species in the *dunni* group to identify proteins driving this incompatibility. These assembled genomes are of similar quality and composition as other higher quality genomes in the *Drosophila* subgenus (Zhou *et al.* 2012; Zhou and Bachtrog 2015; Gramates *et al.* 2017; Hill *et al.* 2019). We also estimated levels of divergence between species and find several structural differences in reproduction genes which may be associated with incompatibilities. Finally, we estimated rates of evolution across these genomes and identify several pathways of groups of genes of interest diverging between species (particularly between *D. nigrodunni* and *D. arawakana*), including rapid divergence in immune pathways and in seminal fluid proteins.

## Materials and methods

### *Drosophila* stocks, husbandry and inbreeding

We obtained stocks for *D. arawakana* (stock number: 15182-2260.00)*, D. dunni* (stock number: 15182-2291.00)*, D. nigrodunni* (stock number: 15182-2311.00) and *D. similis* (stock number: 15182-2321.00) from the National Drosophila Species Stock Center. Importantly, we note that there is reason to suspect that our *D. similis* line may actually be a subspecies of *D. dunni* that is mislabeled at the Stock Center. First, after acceptance of the manuscript, Hope Hollacher (personal communication), who did some of the pioneering work with these species, let us know that she suspected mislabeling. Second, previous phylogenetic analysis of the four species would suggest *D. similis* should be more divergent from *D. dunni* than it appears in this study (Brisson *et al.* 2006). However, we maintain our original labeling as *D. similis* throughout. Each species was maintained on standard instant fly food (Formula 4-24, Carolina Biological Supply Company, Burlington, NC) in an incubator at 23 °C. Before experiments, we inbred for three generations. Specifically, we established 10 separate crosses between individual pairs of a male and female for each species and chose a single successful cross per generation. We then repeated this for two further generations. We then randomly chose one of the successful inbred vials of flies to work with for each species for the remainder of the experiments described.

### Experimental crosses within and between species

We performed initial crosses in all pairwise combinations of species, for both directions of the cross, as well as within species crosses, to confirm previous assessments of between species viability (Heed 1962; Wilder and Hollocher 2003).

For each species we cleared vials of adults at 9:00AM US central time and then collected any emerged adults in 3-hour intervals, separating collected flies by sex. We then used these virgin flies to mate all species in pairwise combinations individually in 3 replicates. For each replicate we mated 10 males with 10 females (all aged 2–3 days) for 5 days (Wilder and Hollocher 2003; Cenzi De Ré *et al.* 2010). Following the 5-day mating period and removal of the parents, we collected offspring every day for 30 days. After aging virgin F1 offspring for 3 days, we mated 10 F1 virgin offspring to 10 flies of the opposite sex from their paternal species, to assess the fertility of the F1 flies. As *D. arawakana* was infected with *Wolbachia*, we sought to cure all species of any bacteria which may affect crosses. We created sublines of each species raised on food containing tetracycline-hydrochloride (0.05 mg/ml) for three generations. We then extracted DNA from females of each strain and tested for *Wolbachia* using PCR (wsp-81F (5′-TGGTCCAATAAGTGATGAAGAAAC-3′), wsp-691R (5′-AAAAATTAAACGCTACTCCA-3′), under the following cycling conditions: 94 °C for 4 minutes, followed by 30 cycles of 40 seconds at 94 °C, 40 seconds at 55 °C, 60 seconds at 72 °C and a final extension step of 72 °C for 10 minutes) (Zhou *et al.* 1998). We then repeated experimental crosses, as described above, with the tetracycline cured strains.

We assayed female survival for *D. arawakana, D. dunni, D. nigrodunni* and *D. similis* in unmated flies and following mating, in both uncured and tetracycline cured flies. We considered a cross to be conspecific if we mated within species (*e.g. D. dunni x D. dunni*) and a cross to be heterospecific if we mated with the most closely related species where fertile hybrids were found in previous crosses (*e.g. D. dunni × D. similis* and *D. arawakana × D. nigrodunni*). For these crosses we established 5-15 vials of 10 males and 10 females of the given species (with no males when measuring virgin females), all aged 2-3 days. We then recorded the survival of females every day (checking at 10:00 AM US central time) for 30 days, flipping the flies onto new food every 3-4 days and removing males after the first 5 days. We fit a survival curve across the total data for each cross-type using SurvMiner (Kassambara *et al.* 2017) in R (Team 2013) and used Cox’s Proportional Hazards Model to identify significant differences in survival between sets of crosses. For the initial crosses we used the following model:

Survival (days post mating) ∼ female species  * male species (if any)+vial.

We set the reference level as the conspecific cross (*e.g. D. arawakana ♂ x D. arawakana* ♀) and looked for significant differences from these for interaction terms to determine if unmated females (*e.g. D. arawakana* ♀ not mated) or heterospecifically crossed females (*e.g. D. arawakana ♂ x D. nigrodunni* ♀) show significant differences from the conspecific cross. To consider the effect of *Wolbachia* infection on these crosses, we repeated these initial crosses alongside the same crosses with *Wolbachia* cured flies (cured as described above) and Cox’s Proportional Hazards Model was used to determine the effect of *Wolbachia* on survival, and to test for differences in survival between sets of crosses after accounting for *Wolbachia*:

Survival (days post mating) ∼ female species * male species (if any)+Wolbachia infection+vial.

### Postmating dissection of the female reproductive tract

We collected virgin males and females for tetracycline-cured *D. arawakana* and *D. nigrodunni* as described above and aged them 2–3 days. We then established conspecific and heterospecific experimental crosses for 6 replicates of 10 males and 10 females at 10:00 AM US central time, as well as virgin control females for 6 replicates of 10 females. Following 24 hours of cohabitation, for 3 replicates of each cross, we separated the females for each cross and dissected the reproductive tract. Based on previous work describing the insemination reaction (Patterson 1947; Grant 1983; Markow and Ankney 1988), we scored the reproductive tract for each female, identifying if the female had mated (by the presence of sperm), if the reproductive tract appeared to be swollen (relative to the unmated virgin females) or if the reproductive tract was destroyed or damaged (alongside a swollen tract, if possible to tell). We repeated this scoring for the remaining 3 replicates of each cross 24 hours later (48 hours total). We then compared conspecific and heterospecific crosses for rates of mating and rates of swelling in the reproductive tract.

### Genome sequencing, assembly and annotation

We extracted DNA following the protocol described in (Chakraborty et al. 2016 ) for *D. arawakana, D. dunni, D. nigrodunni* and *D. similis* females. We prepared the *D. dunni* and *D. nigrodunni* DNA as a sequencing library using the Oxford Nanopore Technologies Rapid 48-hour (SQK-RAD002) protocol, which we then sequenced separately using a MinION (Oxford Nanopore Technologies, Oxford, UK) (Jain *et al.* 2016a) (Supplementary Table 1). We also prepared the *D. arawakana, D. dunni, D. nigrodunni* and *D. similis* samples as Illumina libraries with a 300 bp insert size which we sequenced on an Illumina HiSeq4000 to produce 150 bp paired-end reads (Supplementary Table 1)*.* We removed Illumina adapters using Sickle (Joshi and Fass 2011) and trimmed the Illumina sequences using Scythe (Buffalo 2018). For the two MinION genomes, bases were called *post hoc* using the built in read_fast5_basecaller.exe program with options: –f FLO-MIN106 –k SQK-RAD002 –r–t 4 (Jain et al. 2016b). For *D. dunni*, raw reads were assembled using Minimap2 and Miniasm (parameters: -x ava -o nt -t 8) (Li 2016). We then polished using Racon with Oxford Nanopore Technology reads for three iterations and Pilon with Illumina fragment library reads for three iterations (Walker *et al.* 2014). For the *D. nigrodunni* genome, we first used wtdbg2 to assemble the genome (parameters: –t 4 –L 1000) (Ruan and Li 2020). We then created a second assembly using Minimap2. For each, we ran Racon and Pilon for three iterations as described for *D. dunni*, then merged the two *D. nigrodunni* assemblies using Quickmerge (Liu and Yang 2013). Following this, we polished this merged genome using Pilon for four more iterations. Both assemblies were benchmarked using BUSCO (v 3.0.2) and the *Diptera* database (Simão *et al.* 2015).

For *D. similis*, we mapped data to the *D. dunni* genome before Pilon polishing and polished the *D. dunni* genome using *D. similis* data in Pilon for three iterations, to insert *D. similis* variants into the genome. Following these three iterations of polishing, we mapped *D. similis* data to this genome using BWA (Li and Durbin 2009) and SAMtools (Li *et al.* 2009), and called variants using Picard (http://broadinstitute.github.io/picard) and GATK Haplotypecaller (McKenna *et al.* 2010; DePristo *et al.* 2011). We then used BCFtools (Narasimhan *et al.* 2016) to filter these variants, removing calls below a quality threshold of 200 and inserted them into the polished genome. This was repeated for two more iterations to create a *D. similis* alternate genome. The same pipeline was followed for *D. arawakana* mapped to the *D. nigrodunni* genome.

We used the *D. innubila* transcriptome (NCBI project PRJNA524688) (Hill *et al.* 2019) as well as protein databases from *D. innubila, D. virilis*, *D. melanogaster*, and *M. domestica* in MAKER2 (Holt and Yandell 2011) to annotate each genome, including using *RepeatModeler* (Smit and Hubley 2008) in an attempt to correctly assign repetitive regions and retraining a HMM using SNAP following each iteration (Johnson *et al.* 2008). This was repeated for three iterations to generate a GFF file containing gene evidence generated by MAKER2 (Holt and Yandell 2011).

Finally, we identified orthologous genomic regions pairwise for each of the four species examined here to each other and to the *D. innubila* genome using progressiveMauve (Darling *et al.* 2004). We visualized orthologous regions using rCircos (Zhang *et al.* 2013). We attempted to confirm any apparent structural differences based on progressiveMauve by mapping short reads for each species to a different genome and calling copy number differences using Delly (Rausch *et al.* 2012) and dudeML (Hill and Unckless 2019), taking the consensus of the two tools, but favoring the absence of a copy number variant when we found discrepancies between the two tools.

### Assessing the repetitive content across the dunni group

For each genome, we identified the repetitive content *de novo* using RepeatModeler to call the repeats (engine = NCBI) (Smit and Hubley 2013-2015) and RepeatMasker (-gff –gcalc –s) to identify the repetitive regions (Smit and Hubley 2013–2015). We also used dnaPipeTE (genome coverage = 1, sample number = 2, cpu = 4, genome size = 168000000) (Goubert *et al.* 2015) to identify the repetitive content in the short-read data for each species, which we used to make a second map of reference genome repetitive regions using RepeatMasker. For both sets of repeat content assemblies we identified which TE families were shared between species and which were unique to species using blastn (e-value < 10e–5, hsps = 1, alignments = 1). We then identified what proportion of the genome each TE family constituted across species using RepeatMasker (Smit and Hubley 2013–2015).

### Phylogenetic relationship of the *dunni* group to the rest of the *Drosophila* genus

We next sought to estimate the consensus species tree to better place the *dunni* group in the *Drosophila* phylogeny. We chose to assemble a consensus multigene phylogeny as different genes have differing evolutionary histories and so the tree of a single gene may not best reflect the actual species (Mendes and Hahn 2016), We randomly sampled 100 conserved *Drosophila* genes which have orthologs in the human genome to support their conservation across large evolutionary distances for better estimating a phylogeny (genes chosen based on the list of orthologous *Drosophila* genes on FlyBase) (Gramates *et al.* 2017). We then extracted the coding sequences of these genes from our four focal species, as well as from several other *Drosophila* species found from Flybase genome database (Gramates *et al.* 2017) and the NCBI genomes database (Zhou *et al.* 2012; Hamilton *et al.* 2014; Palmieri *et al.* 2014; Zhou and Bachtrog 2015; Kitts *et al.* 2016; Hill *et al.* 2019). We aligned each group of orthologous genes separately using MAFFT (–auto) (Katoh *et al.* 2002) and created a multiple gene super-tree based on the consensus of each gene tree, following 100 bootstraps with PhyML (-b 100 -N 100 -GTR -gamma 8) (Le and Gascuel 2008; Guindon *et al.* 2010). We also generated gene trees for each of the 100 genes independently, following the same protocol. In this case 66 of the 100 trees gave the same topology of the *dunni* group as the total tree, whereas 7 trees had distinct topologies and 27 trees gave the topology of *D. similis* as an outgroup to the other three species, with *D. dunni* a sister to the *D. nigrodunni-D. arawakana* complex.

### Estimating rates of evolution across the *dunni* group

For each gene in the genomes of our four focal species, we identified orthology to each other and to genes in *D. innubila* using blastp (e-value < 0.00001, hsp = 1 alignment = 1) (Altschul *et al.* 1990). We aligned each set of orthologs using PRANK to generate a codon alignment and gene-tree (Löytynoja 2014), as subtle differences between the species tree and gene trees can result in false estimates of divergence (Mendes and Hahn 2016). We then estimated rates of both non-synonymous and synonymous substitutions using codeML (Yang 2007). Specifically, we estimated synonymous divergence (dS), non-synonymous divergence (dN) and the proportion of the two values (dN/dS). We estimated specific rates of evolution along each branch of the *dunni* group and leading into the *dunni* group using *D. innubila* as an outgroup using a free-ratios model (model 1), and compared this to a model for uniform evolution across all branches (model 0) (Yang 2007), choosing the best fitting model using a likelihood ratio test (*p <* 0.05). Finally, we also estimated rates of evolution across the entire *dunni* group phylogeny using codeML (nsites models M7 and M8) (Yang 2007), choosing the best fitting model using a likelihood ratio test (*p <* 0.05). For all genes sets we removed genes that had saturated dS (dS > 1) which could result in a miscalculation of dN and dN/dS, removing 290 ortholog groups in total.

Using the estimated rates of evolution, we then compared the rates of evolution of all genes on the same branch, as well as the rate of evolution of the same gene between branches. For genes of similar levels of synonymous divergence (dS, windows of 0.001 dS, *e.g.* all genes within 0.001 dS of each other) we found the 97.5th upper percentile for dN/dS on each branch and across the total phylogeny. For our analyses we chose to focus on the upper and lower 97.5th percentiles as these values should be higher or lower than 2 standard deviation from the mean, given our normally distributed results (Shapiro–Wilk test *P* > 0.169). For the closely related species pairs (*D. nigrodunni* and *D. arawakana*, *D. dunni* and *D. similis*) we compared measures of dN/dS between species and found the 97.5th upper percentile for dN/dS per species per window of dN/dS for the paired species (0.001, sliding 0.001).

We then took outlier genes (*e.g.* genes above the 97.5th percentile in each category) and looked for enrichments in gene ontology categories compared to non-outlier genes using GOrilla (Eden *et al.* 2009). For GO categories of interest, such as those enriched for duplications or for high levels of dN/dS, we compared dN/dS of genes in these categories to the nearby genomic background. For each gene we extracted nearby genes (within 100kbp up or downstream on the same chromosome), of similar divergence levels on each branch (within 0.01 dS), we found the difference in dN/dS between the median of the background genes and the focal gene. We used a Wilcoxon-Rank Sum test to identify GO categories on each branch with significantly higher (or lower) dN/dS than the background.

Using the annotations of all species and *D. innubila*, we identified genes with more than one copy in one species, relative to all other species. We confirmed this by estimating copy numbers of genes in each species using short read information and dudeML (following the tutorial pipeline for *N* = 1) with the short read information mapped to the genome of the sister species (Hill and Unckless 2019). We then used GOrilla (Eden *et al.* 2009) to identify Gene ontology categories that are enriched for duplicates on specific branches, which we confirmed using PANTHER (Thomas *et al.* 2003).

### Statistics

We used R for all statistics in this analysis (Team 2013), and ggplot2 for data visualization and figure production (Wickham 2016). False-discovery rate (FDR) multiple testing correction was performed in cases when necessary using R (R-Core-Team 2013).

## Results

### The *Drosophila dunni* group shows varying levels of hybrid incompatibility

**Figure 1:**
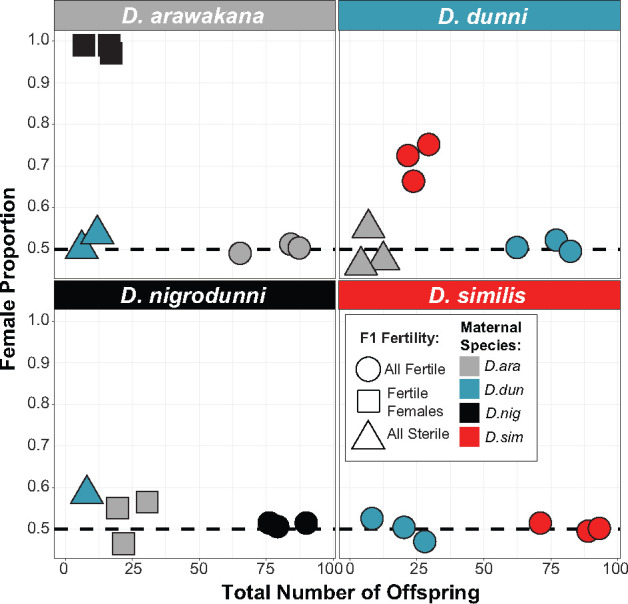
Points show the number of offspring produced in three replicate crosses of 10 females of each species when crossed to males of different species. Boxes are separated by the paternal strain used in each cross. Points are colored based on the maternal strain used in each cross. Point shape shows the state of fertility of F1 offspring, either both fertile (circle), both sterile (triangle) or only females fertile (square). *D. ara* = *D. arawakana, D. dun* = *D. dunni, D. nig* = *D. nigrodunni, D. sim* = *D. similis*. Although we performed all pairwise heterospecific crosses, only crosses which produced offspring are shown on the plot, so fewer than 3 crosses are shown in some cases.

We first surveyed the reproductive divergence between species in the *D. dunni* group. As shown previously (Heed 1962; Wilder and Hollocher 2003), we found that these species have varying levels of hybrid incompatibilities, with some crosses producing viable offspring (Figure 1, *e.g. D. dunni* x *D. similis*) and others producing sterile offspring (Figure 1, *e.g. D. arawakhana* x *D. dunni*) or no offspring (*e.g. D. nigrodunni* x *D. similis*). In keeping with Haldane’s rule (Haldane 1922), some produce sterile males, or no males at all (Figure 1 and Supplementary Table 2, *e.g. D. nigrodunni* x *D. arawakhana*), whereas no crosses produced only males or only fertile males. Despite divergence on levels comparable to the *D. melanogaster* subgroup (Supplementary Figure 1 and Supplementary Table 3), there are no characterized inversions between species (Stalker and Streisinger 1953; Cordeiro *et al.* 2014), allowing differences across the species group to be investigated with a higher resolution than the *D. melanogaster* group allows. Given the variety in levels of divergence and isolation between species (Figure 1, Supplementary Figure 1), we next examined the patterns of divergence between species. Our focus is on the two hybrid crosses which produce some fertile hybrid offspring, such as with *D. nigrodunni* and *D. arawakana*, in which one direction of the heterospecific cross produces only female offspring (Figure 1).

### *Drosophila arawakana* males reduce the lifespan of *D. nigrodunni* females

Females and males within a species are able to coevolve: if males evolve reproductive strategies (including seminal fluid proteins) that harm their mates (Chapman 2001; Ravi Ram and Wolfner 2005; Ahmed-Braimah *et al.* 2017), females can then evolve new ways to tolerate those strategies (Haerty *et al.* 2007). This is not true between species, so we expect that a history of reproductive conflict within species might be evident in heterospecific crosses ( Phadnis and Orr 2009). Females mating with heterospecific males may be unequipped to deal with the mating strategies those males have evolved. Such a mismatch could result in reduced survival after mating, or increased stress response in females (Ahmed-Braimah *et al.* 2020). To search for evidence of sex bias factors causing reduced postmating survival, we established crosses between pairs of species produced some fertile offspring (*D. nigrodunni ♀ x D. arawakana ♂, D. arawakana ♀ x D. nigrodunni ♂, D. similis ♀ x D. dunni ♂, D. dunni ♀ x D. similis ♂*, Supplementary Table 2). We also established matched crosses within species, and a matched control of virgin (unmated) females. For each cross we recorded the survival of females following 5 days of mating.

**Figure 2:**
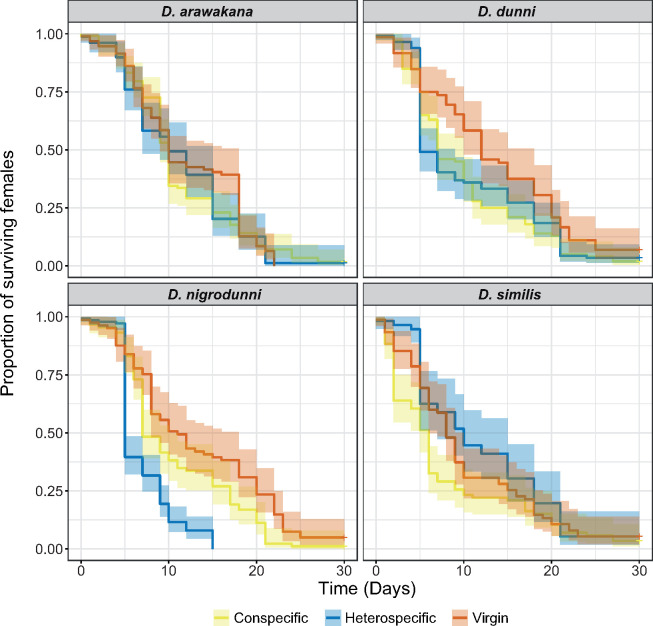
Survival of females postmating. The proportion of surviving females over time after mating, for each species used in each cross, compared to virgin female survival. Boxes show the survival of females separated by their species, and colored based on the females being unmated (virgin, red), conspecific crossed (yellow, crossed to a male of their own species), heterospecific crossed (blue, crossed to a male of a different species). In the case of heterospecific crosses, *D. arawakana* is only crossed to *D. nigrodunni* and *D. dunni* is only crossed to *D. similis*. The shaded regions of each line represent the standard error of the survival curve.

In all cases, and consistent with studies in *D. melanogaster* (Chapman *et al.* 1993; Wigby and Chapman 2005), unmated flies generally survive longer than mated females, though not always significantly (Figure 2, Cox’s Proportional Hazards Ratio = 1.42, *z* = 3.868, *P =* 0.00011). The heterospecific crosses showed no difference from the conspecific crosses for *D. similis* and *D. dunni* (Figure 2, Cox Hazards Ratio = 0.931, Ratio *z* = –0.488, *P =* 0.62545), though *D. similis* heterospecifically mated females lived longer than conspecifically mated females (Figure 2, Cox Hazards Ratio = 1.35, *z* = 2.153, *P =* 0.03134). In contrast, when *D. nigrodunni* females are crossed to *D. arawakana* males, females have significantly decreased survival compared to conspecific crosses and virgin females (Figure 2, Cox Hazards Ratio = 0.489, *z* = –3.360, *P =* 0.00078), the same cross which also produced only female offspring (Figure 1), potentially also caused by the incompatibility of sex bias factors .

As the *D. arawakana* strain examined was infected with *Wolbachia* and the *D. nigrodunni* was not, we cured all strains of bacteria using tetracycline-hydrochloride and repeated the survival assays. All females in this second block have reduced survival compared to the original survival assay, (Supplementary Figure 2, Cox Hazards Ratio = 0.783, *z* = –5.654, *P =* 1.56e–08), suggesting a difference in the two experiments that could be attributed to tetracycline-hydrochloride exposure or a simple block effect. In the tetracycline-hydrochloride exposed flies, we again found reduced survival in the *D. nigrodunni* ♀ *x D. arawakana ♂* cross compared to the conspecific crosses (Supplementary Figure 3, Cox Hazards Ratio = 0.671, *z* = = –3.815, *P =* 0.000136), refuting the idea that *Wolbachia* is causing the postmating lethality observed.

### Swelling in the reproductive tract may be associated with the reduced female survival and reduced number of hybrid offspring

Other studies have highlighted a reaction between the seminal fluid of one species with the environment of the female reproductive tract in the other species, called the insemination reaction (Patterson 1947; Grant 1983; Markow and Ankney 1988). In the hours following mating, the reproductive tract swells, and, in some cases, this results in damage to the reproductive tract (Patterson 1947).

Given the reduced survival of *D. nigrodunni* females following mating with *D. arawakana* males and the reduced number of hybrid offspring, we hypothesized that an incompatibility between the diverged seminal fluid proteins and the heterospecific reproductive tract could cause the swelling of the reproductive tract which reduces female survival and the chance of producing hybrid offspring (Patterson 1947; Markow and Ankney 1988; Knowles and Markow 2001; Markow and O'Grady 2008). This swelling may be caused by a reaction between factors in the seminal fluid and the reproductive tract of the female *Drosophila*, because of the toxic nature of several components in the seminal fluid (Patterson 1947; Markow and Ankney 1988; Knowles and Markow 2001; Markow and O'Grady 2008; Bono *et al.* 2011).

**Figure 3:**
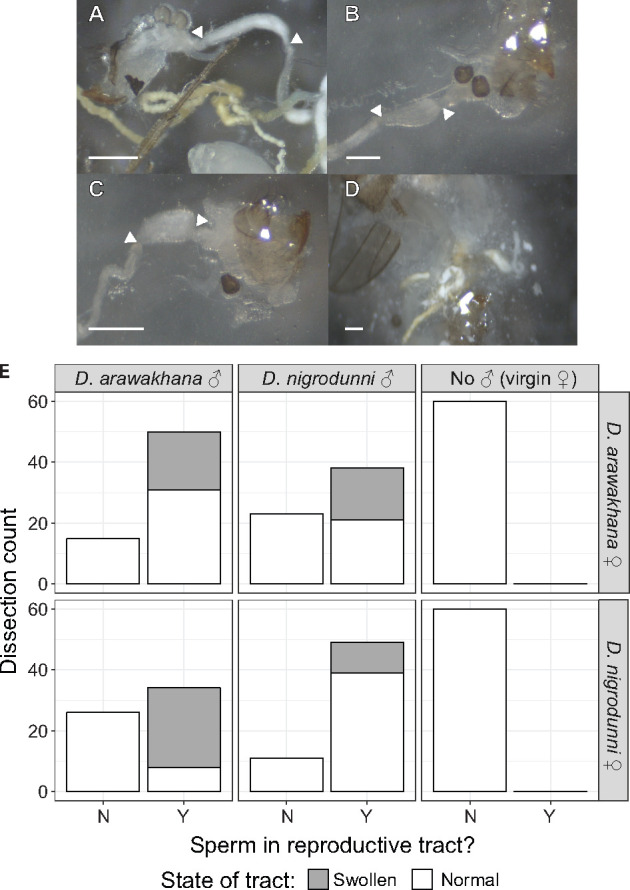
Abnormal insemination reactions may be responsible for reproductive isolation. (A–D) Dissections showing differing conditions of the female reproductive tract. When applicable, arrows label the start and end of same section of the oviduct between dissections. Ovipositors and scale bar also shown for scale. (A) Normal oviduct containing sperm. (B) Normal oviduct with no sperm. (C) Swollen oviduct containing sperm. (D) Ruptured oviduct in sample with sperm. (E) Plots summarizing rate of mating, and the outcome of mating on the reproductive tract in crosses within and between *D. arawakana* and *D. nigrodunni*. Plots are separated by the male involved in the cross (columns) and the female involved in the cross (rows), with plots scoring the number of females with sperm in the reproductive tract, and if the tract was normal or swollen/damaged.

We established experimental crosses within and between *D. arawakana* and *D. nigrodunni*. Then, 24 and 48 hours after crossing we dissected the females to identify whether sperm was present in the female reproductive tract (Figure 3, A and B), and score for abnormal reproductive tracts consistent with the insemination reaction (Figure 3, C and D). Interestingly, there were no significant differences between the number of mated females 24 and 48 hours after establishing crosses (Logistic regression: sperm presence ∼ collection date: *z* = 1.285, *P =* 0.198873), but we did score significantly fewer mated females in heterospecific crosses versus conspecific crosses, suggesting that fewer successful matings occurred in hybrid pairs and some form of premating isolation between these geographically separated species (Logistic regression: sperm presence ∼ cross type: *z* = –2.948, *P =* 0.00319). In several mated females when compared to virgin females, we found a swelling of the reproductive tract consistent with the insemination reaction (Figure 3C). Exclusively in several heterospecifically crossed females, we also saw damaged and destroyed reproductive tracts (Figure 3D). We found a significant excess of swollen/damaged tracts in heterospecifically mated *D. nigrodunni* compared to conspecific controls (Figure 3E, Logistic regression: swollen tract ∼ *D. nigrodunni* cross type: *z* = 4.723, *P =* 2.32e–06). We found swollen tracts in *D. arawakana* females and found no difference between heterospecific and conspecific females (Figure 3E, Logistic regression: swollen tract ∼ *D. arawakana* cross type: *z* = 0.493, *P =* 0.622162). This swelling in the conspecific crosses may be caused by toxic factors in the seminal fluid of *D. arawakana*, as seen in other *Drosophila* subgenus species (Markow and Ankney 1988; Knowles and Markow 2001; Markow and O'Grady 2008).

### Genes involved in copulation and immune defense have high rates of divergence between species

We reasoned that these incompatibilities between species could be caused by a divergence in reproduction proteins. Previous work has suggested that females may be susceptible to severe insemination reactions following hybrid matings (Markow and Ankney 1988; Knowles and Markow 2001). Specifically, that there is an arms race between sexes to block/unblock the female reproductive tract and that females of other species have not evolved to suppress these reactions (Markow and Ankney 1988; Knowles and Markow 2001; Markow and O'Grady 2008). Based on this, we sought to examine the levels of divergence and identify rapidly evolving genes between species which could be involved in this intersexual conflict. We sequenced, assembled and annotated the genomes of each species involved in our survey (see Materials and Methods), producing two high quality genomes with high synteny to each other and to *D. innubila* (Supplementary Tables 1 and 4 and Supplementary Figure 4A), and two assemblies derived from these *de novo* assemblies. The two *de novo* assemblies had high BUSCO scores (*D. dunni* scored 93.9%: 2627 complete, 79 fragmented and 93 missing out of 2799 total; *D. nigrodunni* scored 97.3%: 2721 complete, 37 fragmented and 41 missing out of 2799 total). Consistent with previous findings that failed to detect inversions within the *dunni* group (Cordeiro *et al.* 2014), we found no large structural rearrangements between genomes, and no evidence of fixed inversions between species in the *dunni* group (Heed 1962; Cordeiro *et al.* 2014), though we do find several inversions between the next closest whole genome available, *D. innubila* on Muller elements B, C and D (*D. nigrodunni* shown in Supplementary Figure 4B). We annotated the *dunni* group genomes using a transcriptome from *D. innubila* in MAKER (Holt and Yandell 2011) and found between 10752 and 11581 genes in the assembled genomes of each species, most of which show orthology to previously identified genes in *D. virilis, D. melanogaster* or *D. innubila* (Supplementary Table 5) (Hill *et al.* 2019).

When examining the repetitive content of each species, we found an expansion of Helitrons and long terminal repeat retrotransposons (LTRs) along the *D. dunni/D. similis* branch, resulting in higher transposable element (TE) content in these two species compared to *D. nigrodunni/D. arawakana* (Supplementary Figure 5). We also found species-specific expansions of satellites, particularly in *D. arawakana* and *D. nigrodunni*, where ∼4% of the genome appears to be satellite sequences exclusive to that species (Supplementary Figure 5).

**Figure 4:**
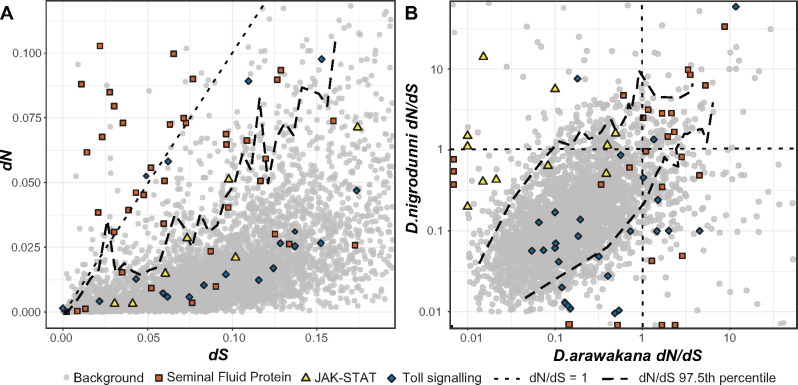
Rates of evolution across the *Drosophila dunni* phylogeny, showing non-synonymous divergence versus synonymous divergence across (A) the whole phylogeny and (B) comparing the proportion of non-synonymous to synonymous divergence between *D. nigrodunni* and *D. arawakana*. JAK-STAT, Toll and seminal fluid proteins are highlighted due to their enrichments in one or the other species.

We identified orthologous genes across species using BLAST (Altschul *et al.* 1990) with *D. innubila* as an outgroup when possible. For each group of orthologous genes, we identified the proportion of synonymous (dS) substitutions and amino acid changing, nonsynonymous substitutions (dN) (per possible synonymous or nonsynonymous substitution, respectively) occurring on each branch of the phylogeny using codeML (branch-based approach) (Yang 2007). We also estimated these substitution rates across the entire *dunni* group phylogeny (Figure 4A, sites-based approach, M7 and M8) (Yang 2007). This allowed us to calculate dN/dS to identify genes showing signatures of rapid or unconstrainted evolution on any branch of the phylogeny, or across the entire tree (Figure 4A). For the dN/dS estimates on each branch, we identified genes in the upper 97.5th percentile for dN/dS in windows of 0.01 dS. dN/dS in *D. nigrodunni* is significantly correlated with dN/dS in *D. arawakana* (Figure 4B), as well as in all other pairwise species comparisons (Figure 4, Pearson’s correlation coefficient = 0.844, t = 7.3774, df = 7569, *P =* 1.786e–13). We find several of the same proteins (or proteins in the same functional group) are rapidly evolving across the entire phylogeny (Figure 4A and Supplementary Table 6). Primarily, copulation proteins (specifically a subset of these, seminal fluid proteins) are overrepresented among the most rapidly evolving genes on every branch of the *dunni* group phylogeny (Supplementary Table 6, *p <* 0.05 after FDR multiple testing correction). Although not significant outliers, we also find that immune recognition proteins, antiviral RNA and piRNA pathways are also rapidly evolving in some species, consistent with arms races between the species and their parasites (Supplementary Table 6).

Rapidly evolving genes may provide clues into the selective forces acting on species since their divergence. For the main species pairs of interest (*e.g. D. nigrodunni* and *D. arawakana*) we identified genes in the upper 97.5th percentile for windows of dN/dS in the other species, to find genes rapidly evolving in one species but not the other (Figure 4B). As expected, copulation-associated proteins were in the upper 97.5th percentile for both species, whereas genes in the Toll immune pathway are rapidly evolving in *D arawakana* but not *D. nigrodunni*, conversely the JAK-STAT immune pathway is rapidly evolving in *D. nigrodunni* but not *D. arawakana* (Supplementary Table 6, Figure 4B). These results suggest each species may differ in their pathogen pressure, resulting in context dependent immune evolution, as seen elsewhere in the *Drosophila* subgenus (Obbard *et al.* 2009; Hill *et al.* 2019).

**Figure 5:**
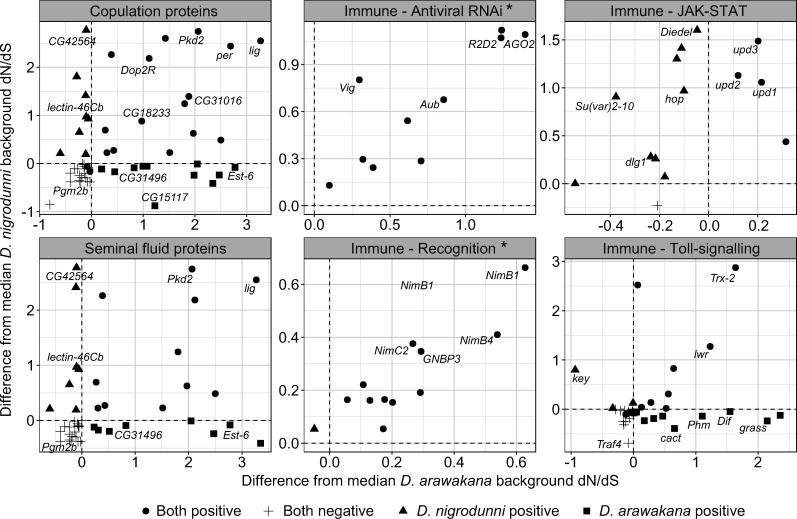
Difference of dN/dS between focal genes in specific functional categories and their nearby background genes, focusing on copulation genes and immune genes. Different copulation proteins and seminal fluid proteins (SFP, a subset of copulation proteins) are rapidly evolving between *D. nigrodunni* and *D. arawakana.* These gene categories are assigned based on FlyBase gene ontology list and refer to genes which have previously been shown to play a role in reproduction. A selection of genes in each category is labeled by name in each plot. Please note the different scales on each axis and plot. Plots are labeled with a * if we find a positive correlation between the two axes (*P <* 0.05).

We sought to confirm the rapid evolution of reproductive pathways and immune pathways after controlling for the background rate of evolution. We found the difference between dN/dS for each immune and reproductive gene and genes at neighboring loci on the chromosome (within 100kbp), of similar levels of divergence (+- 0.01 dS). We found significantly elevated rates of evolution of antiviral proteins, copulation proteins and a subset of these, seminal fluid proteins, across the entire phylogeny (Figure 5, one-sided T-test mu = 0, *P =* 0.0434). We also found a significant correlation between the rates of evolution on the *D. arawakana* and *D. nigrodunni* branches for antiviral genes (Pearson’s correlation = 0.795, *t* = 2.163, *P =* 0.0288), immune recognition genes (Pearson’s correlation = 0.877, *t* = 5.791, *P =* 0.000175) and piRNA genes (Pearson’s correlation = 0.659, *t* = 3.506, *P =* 0.00292). The highest average rate of evolution in our survey occurred in seminal fluid proteins on the *D. nigrodunni* and *D. arawakana* branches (Figure 5, one-sided *t*-test, mu = 0, *p <* 0.05). Consistent with previous results before controlling for background evolution, we find elevated rates of evolution of the Toll signaling pathway in *D. arawakana*, and JAK-STAT in *D. nigrodunni* (Figure 5). Interestingly, when comparing the specific genes rapidly evolving between *D. nigrodunni* and *D. arawakana*, copulation and seminal fluid genes are mostly evolving at different rates between species (Figure 5), whereas the other rapidly evolving genes are consistent between species (Figures 4B and 5). Consistent with this, we did not find a correlation between measures of dN/dS in *D. arawakana* and *D. nigrodunni* copulation genes (Pearson’s correlation = 0.187, *t* = 1.417, *P =* 0.162), seminal fluid proteins (Pearson’s correlation = 0.0341, *t* = 0.224, *P =* 0.823), JAK-STAT (Pearson’s correlation = 0.185, *t* = 0.625, *P =* 0.545) or Toll-signaling proteins (Pearson’s correlation = 0.450, *t* = 1.334, *P =* 0.224). This could suggest that distinct copulation/seminal fluid proteins (and immune pathways) are important in each species, as different proteins are rapidly evolving in the different species (Figure 5), and may even suggest (though unlikely) a molecular divergence in the roles these proteins are playing in each species (Haerty *et al.* 2007).

Using orthology to *D. innubila*, we also identified duplications relative to these two species in each *dunni* group genome, and specific to each species. Consistent with the estimates in rates of evolution, we found enrichments of duplications in cell motility and copulation (specifically premating behavior) across the entire phylogeny (Supplementary Figure 6 and Supplementary Table 7). We also found enrichments of duplications in Toll signaling genes in *D. arawakana* (*P =* 0.000569, enrichment = 5.44). Overall this suggests that the pathways showing elevated levels of nucleotide divergence (namely Toll signalling and copulation genes) also have more copy number variation between species than expected.

## Discussion

*Drosophila* species have served as prominent models in genetics research, including in understanding the divergence between populations and the evolution of species (Coyne and Orr 1989; Orr 2005). This is facilitated by the extensive genetic tools available in the species group to identify the genetic basis of reproductive isolation, both prezygotic and postzygotic (Orr 2005; Phadnis and Orr 2009; Hales *et al.* 2015; Turissini *et al.* 2018). Many islands contain endemic species of *Drosophila* with differing levels of isolation. For example, the island endemics in the *D. simulans* complex (Cabot *et al.* 1994; Kliman *et al.* 2000; Matute and Ayroles 2014), with *D. mauritiana, D. simulans* and *D. sechellia* have served as a rich system for understanding reproductive isolation (Cabot *et al.* 1994; Kliman *et al.* 2000). Like the *D. simulans* complex, the *D. dunni* species subgroup has radiated across a chain of islands (Heed 1962), though with easier to define species relationships than is seen in the *simulans* subcomplex (Cabot *et al.* 1994; Kliman *et al.* 2000; Matute *et al.* 2014). Due to the recent radiation of this group, many species pairs in the *dunni* subgroup produce offspring (Stalker and Streisinger 1953; Heed 1962), some of which are fertile, and so provide a potentially useful model system for dissecting the genetics of reproductive isolation.

Here, we assessed the extent of hybrid incompatibilities between species of the *dunni* subgroup, focusing on postmating-prezygotic incompatibilities. We then sequenced and assembled the species genomes to identify highly divergent and rapidly evolving genes which could be involved in these incompatibilities. Between *D. nigrodunni* and *D. arawakana*, we found elevated divergence of several immune system pathways, as well as divergence in genes involved in copulation. This rapid evolution of copulation genes (primarily seminal fluid proteins) fits with the reduced survival of females following insemination by a heterospecific male. Consistent with the divergence in the seminal fluid proteins, we found evidence of swelling of the reproductive tract (Knowles and Markow 2001), and a decrease in hybrid mating compared to within species, suggesting both pre and postmating prezygotic incompatibilities. We also found several instances of postzygotic incompatibilities between species, including sterility and male inviability (Figure 1).

Most of the striking differences appear when comparing *D. nigrodunni* and *D. arawakana* (Figures 1–5). This pair is slightly less diverged than other pairings within the group (Supplementary Figure 1 and Supplementary Table 3) and are allopatrically separated (Heed 1962; Wilder and Hollocher 2003), allowing for the neutral accumulation of substitutions with a reduced chance of introgression (Coyne and Orr 1989; 2004). Due to this reduced divergence and reduced incidence of incompatibilities (Orr 1995; Welch 2004), we may have caught this species pair at the opportune time where these hybrid incompatible effects are visible (unlike the *D. dunni* and *D. similis* pair which are less diverged, Supplementary Table 3), whereas other species pairs are too far diverged (Figure 1).

The functional annotation of the more diverged genes may also provide us with clues as to how these species are diverging. For example, we identified divergence in the copy number for male mating behavior genes between *D. arawakana* and *D. nigrodunni* (Supplementary Figure 6), and female mating behavior genes are enriched in the upper 97.5th percentile for dN/dS across the whole *dunni* group phylogeny (Supplementary Table 6, enrichment = 15.44, *P =* 0.000113). These accumulating differences may cause a divergence in premating behavior, resulting in the reduced rate of hybrid matings scored (Figure 3). We also see no difference in the proportion of hybrid matings after 24 hours and 48 hours, suggesting that in these cases, if a female has rejected all males, she may not change her mind later, supporting the evolution of some pre-mating isolation between species, despite isolation (Coyne and Orr 2004; Gourbière and Mallet 2010; Turissini *et al.* 2018). Hybridization between island-endemic flies separated by ∼500 kilometers of ocean may be unlikely (Coyne *et al.* 1982), but selection against hybridization between our focal species and other *dunni* group species may have led to the evolution of reinforcement against heterospecific mating (Gourbière and Mallet 2010; Turissini *et al.* 2018). Alternatively, this pre-mating isolation could have evolved due to the neutral divergence between the two species (Orr and Turelli 2001; Turissini *et al.* 2018). We also found seminal fluid proteins are rapidly diverging between species (Figures 4 and 5) and found an increased incidence of swollen and deformed reproductive tracts, consistent with an insemination reaction-like effect and a toxic incompatibility between the seminal fluid proteins and their environment (Figures 2 and 3) (Markow and Ankney 1988; Knowles and Markow 2001). Previous work has highlighted that male expressed genes with the fastest rates of evolution are more likely to contribute to paternal gametic incompatibility (Ahmed-Braimah *et al.* 2017), which may also be the case here. In fact, studies in other species have also identified postmating-prezygotic incompatibilities as a major cause of isolation between species, even in cases with some gene flow (Gavrilets and Waxman 2002; Gavrilets 2003; Larson *et al.* 2012; 2013; Ahmed-Braimah 2016; Turissini *et al.* 2018). A recent study identified the upregulation of the JAK-STAT pathway (a stress response pathway) in *Drosophila* females following heterospecific mating, likely due to the negative effects of the accessory gland proteins (Ahmed-Braimah *et al.* 2020). The rapid evolution of JAK-STAT proteins in *D. nigrodunni* may also be due to this species requiring a well-adapted stress response pathway, given its negative reaction to heterospecific matings (Figures 1–3).

Focusing on specific copulation proteins, we found several proteins are rapidly evolving in all species (Supplementary Table 6). *painless*, a transporter protein, is in the upper 97.5th percentile for dN/dS in all four species analyzed. *painless* plays a role in the pre-mating sexual receptivity of females, and incompatible hybrid interactions with this protein may explain the reduced success in hybrid mating (Sakai *et al.* 2009). Similarly, *lingerer* is also rapidly evolving in *D. arawakana* and *D. nigrodunni*, a protein thought to act as a controller for copulatory organs during courtship and so may affect hybrid mating success (Kuniyoshi *et al.* 2002). We also found several seminal fluid associated proteins are evolving exclusively in one species, such as *Esterase-6* and *Muscleblind* in *D. arawakana* or *Lectin-46Cb* in *D. nigrodunni* (Figure 5 and Supplementary Table 6)*.* Inferred through mutant phenotypes, these proteins appear to regulate female receptivity postmating in *D. melanogaster* (Chapman 2001; Bloch Qazi *et al.* 2003; Ravi Ram and Wolfner 2005). Though the insemination reaction is not seen in *D. melanogaster* (Alonso-Pimentel et al. 1994), it is not unreasonable to assume these proteins may play a role in it in other species and could cause the postmating phenotypes we see here (including the toxic effects in hybrid crosses, Figure 3).

There are several potential possible causes for the rapid evolution of copulation proteins seen in this study. The first, as mentioned above, is due to a reproductive conflict between males and females (Markow and Ankney 1988; Knowles and Markow 2001). As the two sexes benefit from different outcomes of reproduction, each sex will in turn evolve methods to prevent or suppress the reproductive goals of the other sex, resulting in an arms race scenario (Knowles and Markow 2001). Consistent with this, we found rapid evolution of the seminal fluid proteins, the main tool in male sexual conflict (Figure 4) and seminal fluid induced swelling of the female reproductive tract (Figure 3) (Alonso-Pimentel *et al.* 1994; Ravi Ram and Wolfner 2005). The second possible cause of divergence here is reinforcement (Gourbière and Mallet 2010). The reduced hybrid mating coupled with postmating isolation seen between several species may imply that reinforcement may drive divergence, though the species studied here are allopatric, so such reinforcement would have to have evolved prior to geographic isolation (Heed 1962). Relaxed selection could also be occurring if sexual selection is less important in these species (Dapper and Wade 2020). We would expect reduced polymorphism and elevated non-synonymous substitutions weighted by non-synonymous polymorphism if an arms race was occurring between species (McDonald and Kreitman 1991; Messer and Petrov 2013), but elevated polymorphism if relaxed selection was occurring (Dapper and Wade 2020). Unfortunately, polymorphism information is not currently available for our focal species to help determine the main force driving evolution.

Several of the functional gene categories identified in this study as highly divergent between species are also promising regions for future study, particularly when focusing on immune evolution. Our findings are consistent with other studies that find immune proteins are more rapidly evolving than background genes (Sackton *et al.* 2007; Obbard *et al.* 2009; Shultz and Sackton 2019), consistent with an arms-race between the host and its pathogens. However, in the species studied here, we find several cases of species-specific rapid evolution of an immune pathway, such as the rapid evolution of JAK-STAT in *D. nigrodunni*, specifically the genes *Diedel*, *lingerer* and *Socs36E* (Figures 4 and 5). As the general stress response pathway, the JAK-STAT pathway is activated following mating in several *Drosophila* (Hoffmann 2003; Ahmed-Braimah *et al.* 2020), suggesting it may be involved in reproductive conflict in the *dunni* group, resulting in its rapid adaptation. Alternatively, as immune pathways are constantly evolving in response to their pathogens, this difference could be explained by differences in immune pathogens in this species group (Sackton *et al.* 2007; Unckless *et al.* 2016; Hill *et al.* 2019). Hypothetically, the lack of any substantive natural Gram-negative bacterial pathogens in *D. dunni* would result in a lack of rapid evolution (and divergence) in the IMD pathway, the immune pathway associated with resisting infection by Gram-negative bacteria. Although a lack of fungal or Gram-positive bacterial pathogens in *D. nigrodunni* could result in the lack of evolution of the Toll pathway, but rampant evolution in *D. arawakana* (Figures 4 and 5).

The repetitive content also appears to be diverging rapidly across this species complex (Supplementary Figure 5). This is commonly seen between diverging species, given the elevated mutation rate/transposition of selfish factors compared to the rest of the genome (Kofler *et al.* 2012; 2015; Adrion *et al.* 2019), and has been implicated in the formation of hybrid incompatibilities for several species (Satyaki *et al.* 2014). Consistent with this we found several species specific TE families in the *dunni* complex. However, we did not find a significant excess of dysgenic ovaries in hybrid females compared to normal females (Fisher’s exact text *p*-value > 0.05 for all cases). Several cases of hybrid incompatibilities caused by differences in TE content results in sterility caused by maternally inherited factors over paternally inherited (as is usually seen). This may be due to the absence of maternally loaded silencing RNAs against specific TEs (Bingham *et al.* 1982; Aravin *et al.* 2007; Brennecke *et al.* 2008). If this were the case, we would expect the hybrid sterility to be in the opposite direction to what we observe, with sterile females (Figure 1, Supplementary Figure 5) (Kidwell *et al.* 1977), and so do not expect the hybrid incompatibilities seen here to be caused by repetitive content. However, this is a simplistic view of the effects of transposon activity on hybrid fertility, given the complex hybrid dysgenesis cases seen in *D. virilis* (Petrov *et al.* 1995; Evgen'ev *et al.* 1997; Erwin *et al.* 2015), and even the complex cases of tolerance to dysgenesis seen in the supposedly simple case in *D. melanogaster* (Kelleher *et al.* 2018), so may require further study to fully understand if TEs play a role in the divergence of the *dunni* complex.

## Conclusions

Overall, our findings suggest that the rapid divergence of reproductive genes may have led to incompatibilities between species in the *dunni* group, such as the insemination reaction associated with reduced female survival. We also found multiple areas for further investigation in the *D. dunni* group, either in immune evolution or continuing to investigate the speciation in this species group, suggesting promise in the future of research for this group.

## Data availability

All data used in the analyses in this study are available at figshare (https://doi.org/10.25387/g3.13865258). Sequencing information and assembled genomes used in this study is available in the SRA BioProject PRJNA661260 (Supplementary Table 1 has details).
